# Chromosome-wide regulation of euchromatin-specific 5mC to 5hmC conversion in mouse ES cells and female human somatic cells

**DOI:** 10.1007/s10577-012-9317-9

**Published:** 2012-10-31

**Authors:** Musashi Kubiura, Masaki Okano, Hiroshi Kimura, Fumihiko Kawamura, Masako Tada

**Affiliations:** 1 Division of Molecular and Cell Genetics, Department of Molecular and Cellular Biology, School of Life Sciences, Faculty of Medicine, Tottori University, Yonago, 683-8503 Japan; 2 Laboratory for Mammalian Epigenetic Studies, Center for Developmental Biology, RIKEN, Kobe, 650-0047 Japan; 3 Graduate School of Frontier Biosciences, Osaka University, Suita, 565-0871 Japan; 4 Chromosome Engineering Research Center, Tottori University, Yonago, Tottori 683-8503 Japan

**Keywords:** DNA methylation, Embryonic stem cells, Tet enzymes, 5-Hydroxymethylation

## Abstract

DNA cytosine methylation (5mC) is indispensable for a number of cellular processes, including retrotransposon silencing, genomic imprinting, and X chromosome inactivation in mammalian development. Recent studies have focused on 5-hydroxymethylcytosine (5hmC), a new epigenetic mark or intermediate in the DNA demethylation pathway. However, 5hmC itself has no role in pluripotency maintenance in mouse embryonic stem cells (ESCs) lacking *Dnmt1*, *3a*, and *3b*. Here, we demonstrated that 5hmC accumulated on euchromatic chromosomal bands that were marked with di- and tri-methylated histone H3 at lysine 4 (H3K4me2/3) in mouse ESCs. By contrast, heterochromatin enriched with H3K9me3, including mouse chromosomal G-bands, pericentric repeats, human satellite 2 and 3, and inactive X chromosomes, was not enriched with 5hmC. Therefore, enzymes that hydroxylate the methyl group of 5mC belonging to the Tet family might be excluded from inactive chromatin, which may restrict 5mC to 5hmC conversion in euchromatin to prevent nonselective de novo DNA methylation.

**Electronic supplementary material:**

The online version of this article (doi:10.1007/s10577-012-9317-9) contains supplementary material, which is available to authorized users.

## Introduction

Epigenetic reprogramming activity in early embryonic cells and primordial germ cells (PGCs) induces conversion and/or replacement of DNA cytosine methylation (5mC) into unmodified cytosine (C) on a genome-wide scale (Feng et al. 2010). Tet methylcytosine dioxygenase belonging to the Tet enzyme family (*Tet1-3*) converts 5mC into 5-hydroxymethylcytosine (5hmC) (Tahiliani et al. 2009). However, it remains unclear whether 5hmC functions as an epigenetic mark or only as an intermediate during the conversion of 5mC to C (Ito et al. 2010; Koh et al. 2011; Dawlaty et al. 2011; Freudenberg et al. 2012; Gu et al. 2011; Williams et al. 2011). Obviously, 5hmC has no role in pluripotency maintenance in embryonic stem cells (ESCs) lacking DNA methyltransferases *Dnmt1*, *3a*, and *3b* (TKO) in the absence of DNA methylation activity (Szwagierczak et al. 2010; Tsumura et al. 2006). A recent knockout experiment showed that the most abundant Tet enzyme, Tet1, is dispensable for the maintenance of pluripotency in ESCs even in the presence of DNA methylation activity (Dawlaty et al. 2011). Genome-wide distribution of 5hmC was previously determined by hydroxymethylated DNA immunoprecipitation, followed by high-throughput sequencing. These studies demonstrated that 5hmC co-localises with H3K4me and H3K27me3, both of which are active euchromatin marks (Ficz et al. 2011; Pastor et al. 2011). Moreover, genome-wide localisation of Tet1 was also determined by chromatin immunoprecipitation, followed by DNA sequencing, and the results revealed that 5hmC accumulated at transcription start sites of CpG-rich promoters and within genes (Williams et al. 2011; Xu et al. 2011). We previously showed that the euchromatic marks were associated with in vitro reprogramming induced by cell fusion between ESCs and somatic cells (Kimura et al. 2004). Thus, Tet enzymatic activity may be associated with active chromatin formation and/or maintenance in ESCs. Active chromosomal regions generally replicate in early S phase of the cell cycle, while inactive chromosomal regions, including constitutive and facultative heterochromatin, replicate in late S. One exception to this is perinuclear heterochromatin, which replicates in mid S (Nakayasu and Berezney 1989; Panning and Gilbert 2005). Based on this, active chromosomal regions can be determined by the chromosomal replication bands called R-bands as regions that are negative for 5-bromodeoxyuridine (BrdU) labeling during late S to metaphase. By contrast, inactive chromosomal regions can be cytogenetically detected by a simple trypsin–Giemsa technique or 4′, 6-diamino-2-phenylindole dihydrochloride (DAPI) staining, which can produce the differential bands called G-bands on the basis of different chromosome structures. G-bands are often used for identification of each chromosome ID.

The major aim of this study was to visualise the intra-chromosomal distribution of 5hmC using a combination of chromosome banding and immunostaining techniques, and to examine how Tet enzymatic activity is regulated across chromosomes in mouse ESCs and human female differentiated cells from induce pluripotential stem cells (diff-hiPSCs). Modification of certain residues on the N-terminus of histone H3 is important for transcriptional regulation, heterochromatin formation, and silencing of viral-derived sequences (Hayashi-Takanaka et al. 2011). We found that 5hmC-enriched regions overlapped with chromosomal regions enriched with H3K4me2/3, but not H3K9me3.

## Materials and methods

### Cell materials

Undifferentiated mouse ESCs derived from male inner cell mass cells named J1 were used as wild-type ESCs. TKO ESCs deficient for *Dnmt 1*, *3a*, and *3b*, which were originally derived from J1 ESCs, were used as a negative control for 5hmC immunostaining (Tsumura et al. 2006). The transgenic ESCs that stably express EGFP fused with the methyl-binding domain of MBD1 (MBD-EGFP-nls) were used for live imaging of 5mC-enriched regions (Kobayakawa et al. 2007). These ESCs were kindly provided by the RIKEN BioResource Center. The human iPSC line A5 was previously established in our group by viral-mediated introduction of the following four factors into human female fibroblasts: *OCT4*, *SOX2*, *KLF4*, and *c-MYC*. Standard procedures were used to culture mouse ESCs. In brief, mouse ESCs were placed onto mitomycin C-treated mouse embryonic fibroblasts and cultured in the mouse ESC medium [DMEM F-12 (Sigma-Aldrich) containing 13 % fetal bovine serum (PAA Laboratories), 10^3^ U/ml LIF (ESGRO, MILLIPORE), penicillin streptomycin, l-glutamine, sodium pyruvate, and 2-mercaptethanol (2-ME)]. Human induced pluripotential stem cells (iPSCs) were placed on mitomycin C-treated STO cells and cultured in iPSC medium (MEM F-12 containing 20 % KSR, penicillin streptomycin, l-glutamine, sodium pyruvate, 2-ME, nonessential amino acid, 0.5 N NaOH, and bFGF). Most of the reagents used for cell culture were obtained from Gibco. Diff-hiPSCs were obtained using a previously described procedure for inducing hepatic progenitors (Zhao et al. 2009). A5 hiPSCs were treated with 10 ng/ml of Activin A and were further cultured with HGF-containing hepatic growth medium.

### Immunostaining for 5hmC and BrdU

**Fig. 1:**
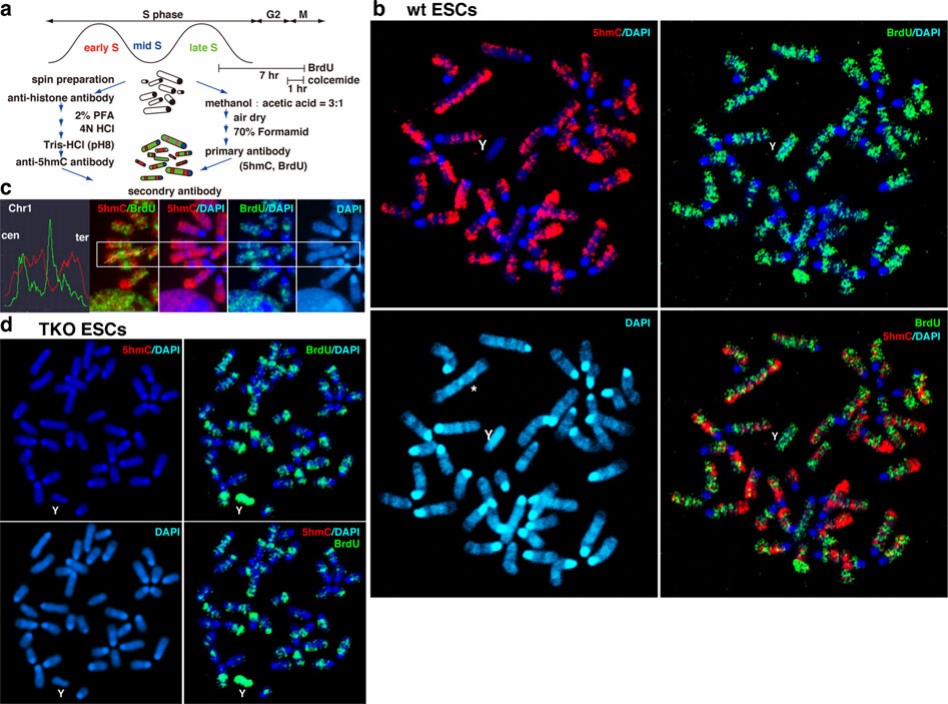
5hmC distribution across mouse chromosomes in ESCs. **a** Experimental schema. **b** Metaphase chromosomes collected by mitotic arrest induced by colcemide that had incorporated BrdU during late S were immunostained for 5hmC (*red*) and BrdU (*green*). 5hmC exhibited R-bands, which were negative for G-bands stained with DAPI (*blue*) and BrdU in a wild-type J1 ESC. Pericentric repeats and Y chromosome (Y) were negative for 5hmC and BrdU. The J1 ESCs often contained an additional Chr8 via centric fusion (*asterisk*). **c** Inverse relationship between 5hmC and BrdU staining on mouse chromosome 1 (Chr1). Two-colour fluorescence analysis of 5hmC and BrdU through the long axis of Chr1 showed primarily opposite patterns. **d** TKO ESCs lacking Dnmts did not show 5hmC enrichment. Most of the metaphase spreads were 30 to 40 μm in size

ESCs and diff-hiPSCs were cultured for 7 h in the presence of 1 mM BrdU (Invitrogen) and treated with 0.3 μg/ml colcemide (Demecolcine, Sigma-Aldrich) for 1 h before collection as shown in Fig. 1a. The cells that had incorporated BrdU during late S were able to enter mitosis through G2 phase in this procedure. Cells were collected by trypsinisation and treated with 0.075 M KCl for 7 min at room temperature. Then, cells were fixed with a mixture of methanol and acetic acid (3:1). Chromosome spreads on glass slides were prepared using the air-drying method. DNA was denatured with 70 % formamide in 2× SSC at 70 °C exactly for 2 min and was immediately dehydrated by 70 % ethanol for 5 min at −20 °C. The samples were then treated with 100 % ethanol for 5 min at room temperature and then dried. Denatured chromosomes were treated with 0.1 % Triton-X in PBS for 10 min at room temperature. After washing with PBS, they were treated with 2 % skim milk in PBS for 30 min at room temperature and then treated with primary antibodies diluted in 2 % skim milk in PBS for 1 h at room temperature. The primary antibodies that the samples were incubated with were mouse monoclonal anti-BrdU antibody (1:100, Abcam) and rabbit polyclonal anti-5hmC antibody (1:500, Active Motif), followed by secondary antibodies Alexa488-mouse IgG (1:500, Molecular Probes, Invitrogen) and Alexa546-rabbit IgG (1:500, Molecular Probes, Invitrogen), respectively. Samples were then washed three times in 0.05 % Tween-20 in PBS. Finally, chromosomes were stained with Prolong Gold antifade reagent containing DAPI (Invitrogen) to detect G-bands. Images of chromosomes were obtained by an LSM780 confocal microscope (Carl Zeiss). For haze reduction-treated images, BIOREVO BZ-9000 (KEYENCE) was used.

### Immunostaining for 5hmC and histone H3

J1 ESCs and TKO ESCs were cultured with 0.3 μg/ml colcemide for 2 h. The cells were collected by trypsinisation and briefly treated with 10 μg/ml cytochalasin B (Sigma-Aldrich) in suspension culture with DMEM. After the cells were collected by centrifugation, they were treated with 0.075 M KCl for 7 min at room temperature. After hypotonic treatment, 1 ml of KCl solution containing 5 × 10^4^ cells was applied to poly-l-lysine-coated 12-mm round coverslips (BD Biosciences) that had been placed at the bottom of the cylindrical tube. Then, the tubes were centrifuged for 3 min at 800 rpm. Chromosome spreads attached to the coverslips were directly treated with 2 % skim milk in PBS for 30 min at room temperature and incubated with primary antibody overnight at 4 °C. The antibodies that had been created and intensively analysed in the Kimura group (Hayashi-Takanaka et al. 2011) were used to stain native chromosomes: mouse monoclonal H3K4me2, H3K9me2, and H3K9me3 (1:1,000). After washing with 0.05 % Tween-20 in PBS three times, the coverslips were fixed with 2 % PFA for 5 min and then washed with PBS two times. Chromosomal DNA was denatured with 4 N HCl for 10 min and then neutralised with Tris–HCl buffer (pH 8.0). The denatured chromosomes were treated with anti-5hmC (1:500) overnight at 4 °C. The series of steps in the procedure are also shown in Fig. 1a. Localisation of MBD-EGFP was visualised by immunostaining with mouse anti-GFP monoclonal antibody (1:200, Santa Cruz Biotechnology) in transgenic MBD-EGFP-nls ESCs. H3K4me3 was detected simultaneously using a rabbit polyclonal antibody (1:1,000, Abcam).

### FISH for mouse Cot1

Chromosome spreads were fixed with a mixture of methanol and acetic acid (3:1) and denatured with 70 % formamide in 2× SSC. Alternatively, samples fixed with 2 % PFA were denatured with 4 N HCl. Mouse Cot1 DNA (Invitrogen) was biotin-labelled with Nick Translation Mix (Roche) and Biotin-16-dUTP (Roche). The labelled DNA was precipitated in ethanol with salmon sperm DNA and dissolved in 100 % formamide. DNA mixture (0.25 μg) was mixed with an equal amount of hybridisation buffer (0.1 % BSA, 10× SSC and 50 % dextran sulfate, 1:2:2). After denaturation, biotin-labelled DNA was placed on denatured chromosomes and covered with a thin film. The DNA hybridisation step was performed overnight at 37 °C in a moisture chamber. Non-hybridised probe DNAs were extensively washed in a mixture of formamide and 4× SSC (1:1) for 15 min at 37 °C, followed by 15-min washes in 2× SSC, 1× SSC, and 4× SSC at room temperature. Biotin-labelled probe DNA was detected by avidin-FITC (1:350).

## Results

In this study, mouse ESCs were treated with BrdU for 7 h, and then colcemide was added 1 h before cells were collected. All of the metaphase chromosomes incorporated BrdU only during late S, and the chromosomal regions replicating in late S were visualised by immunostaining with an anti-BrdU antibody as shown in Fig. 1a. 5hmC-enriched areas were visualised by anti-5hmC antibody staining. Chromosomes were fixed with a mixture of methanol and acetic acid and denatured to expose BrdU and 5hmC from inside of the highly condensed double-stranded DNA. Denaturation is important because the accessibility of 5hmC to the antibody greatly influences the results. DAPI staining was used to identify mouse chromosomes by their G-bands. It was found that BrdU-positive regions replicating during late S in mouse ESCs overlapped with G-bands, but not with the 5hmC signals (Fig.1b). 5hmC signals showed R-bands, but not with BrdU and G-bands on mouse chromosome 1 (Chr1), a representative chromosome (Fig. 1c). Pericentric satellite repeats that replicated in mid-S and displayed G-bands did not overlap with 5hmC (Fig. 1b, c). In addition, 5hmC was also not found on the Y chromosome (Y). Thus, these data suggest that 5hmC accumulate at active regions replicating in early S and overlaps with R-bands in mouse ESCs (Supplement S1).

TKO ESCs lacking *Dnmt1*, *3a*, and *3b* were not enriched with 5hmC due to the absence of 5mC, a major substrate of Tet enzymes (Fig. 1d). Therefore, 5hmC may not be an epigenetic mark that regulates active chromatin formation in the absence of DNA methylation activity in mouse ESCs.

One notable observation was that 5hmC signals were often enriched on only one of the sister chromatids (Fig. 1b). A possible explanation for this is that fully methylated double-stranded DNA (5mC-5mC) is converted into fully hydroxymethylated DNA (5hmC-5hmC) at euchromatin, which turns into hemi-hydroxymethylated DNA (5hmC-C) after the first cycle of DNA replication. Subsequently, the 5hmC-C DNA is converted into 5hmC-C DNA and C-C DNA during the second cell cycle. Then, C-C DNA is converted into 5mC-5mC DNA by a de novo DNA methylase. The sister chromatid exchange caused by spontaneous mitotic recombination might induce the chimeric distribution of 5hmC on chromatids. This is plausible as 5hmC prevents Dnmt1 target recognition (Valinluck and Sowers 2007). Thus, chimeric 5hmC distribution on chromatids might reflect cyclic conversion of 5mC into C via 5hmC.

**Fig. 2:**
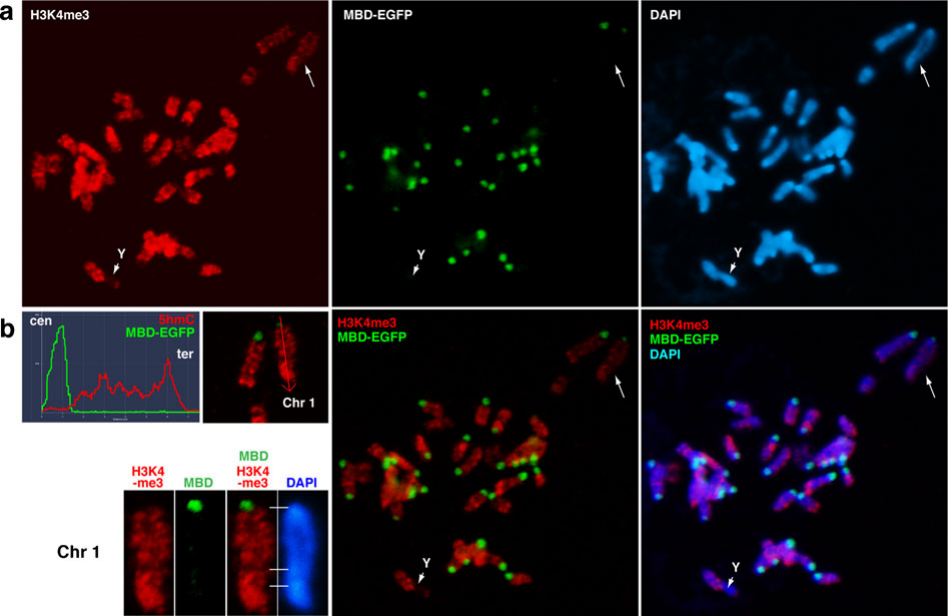
Persistence of 5mC at pericentric repeat regions lacking histone H3K4me3. **a** The MBD-EGFP protein associates with DNA methylated regions. MBD-EGFP accumulated on pericentric repeat regions of chromosomes in transgenic mouse ESCs that ubiquitously express MBD-EGFP (*green*), while H3K4me3 (*red*), an euchromatin-specific mark, was enriched at G-band-negative regions, as shown on Chr1 in (**b**). Y lacked both MBD-EGFP and H3K4me3 enrichment (*arrowhead*)

5mC levels are very low in mouse cells, which make it difficult to detect 5mC with the anti-5mC antibody. We could believe this involved little technical matter with anti-5mC antibody because 5mC localisation was strongly detected with this antibody in chicken cells (unpublished data). To bypass this issue, we monitored 5mC-enriched chromosomal regions by EGFP localisation in transgenic MBD-EGFP ESCs (Kobayakawa et al. 2007). To visualise relationship between EGFP and histone H3K4me3 localisation, chromosome spreads prepared by spin preparation were directly stained with primary antibody and then fixed with PFA. MBD-EGFP was largely enriched on pericentric satellite repeats, but not on the Y chromosome (Fig. 2a). Therefore, 5hmC was not detected on Y due to the absence of preexisting 5mC, while the absence of 5hmC on pericentric satellite repeats is likely due to an active mechanism leading to 5mC maintenance. In addition, the H3K4me3 pattern on Chr1, a representative chromosome, clearly shows that euchromatin enriched with H3K4me3 lacked G-bands (Fig. 2b). The lack of 5hmC enrichment around pericentric satellite repeats is not due to insufficient DNA denaturation. Two types of DNA denaturation procedures were used (Fig. 1a). In both cases, we obtained sufficient fluorescence in situ hybridisation signals using mouse Cot1 probe DNAs containing mouse B1, B2, and L1 repeats, especially at pericentric regions (Supplement S2). Therefore, an active mechanism promoting site-specific exclusion of Tet enzymatic activity may be responsible for the persistence of 5mC at pericentric satellite repeats.

**Fig. 3:**
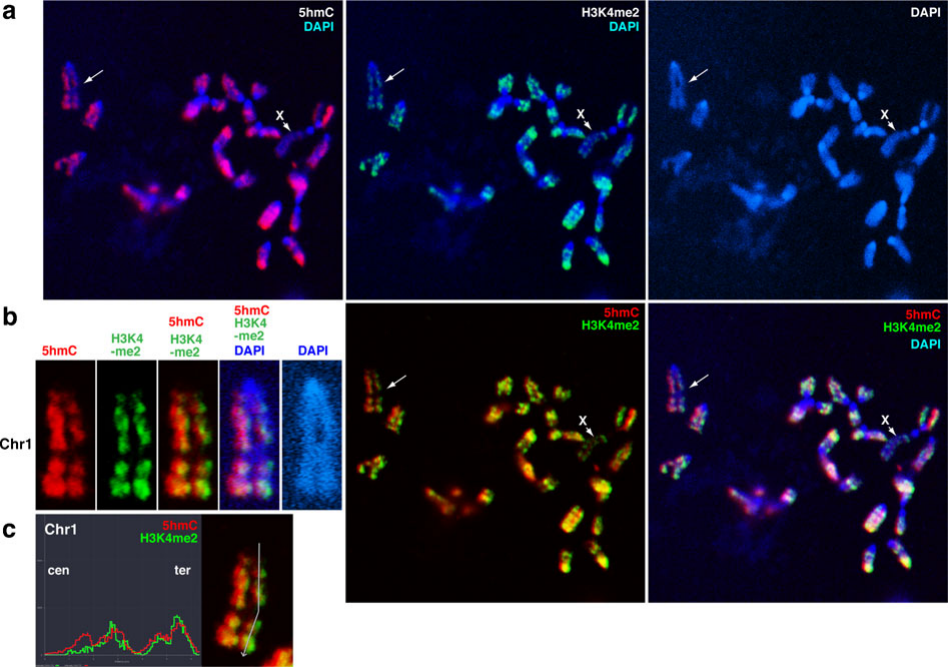
Co-localisation of 5hmC and H3K4me2. **a** Metaphase chromosomes prepared by spin preparation were co-immunostained for 5hmC (*red*) and H3K4me2 (*green*), a euchromatin mark. The merged image is mostly *yellow* due to the co-localisation of 5hmC and H3K4me2 in mouse ESCs. **b** The distribution of two-colour fluorescence through the long axis of Chr1 for 5hmC and H3K4me2 was also similar. **c** Profiles of the flourescent intensity values for 5hmC and H3K9me4 along the line drawn across the long axis of Chr1

Next, to analyse the relationship between 5hmC and specific histone modifications, chromosome spreads were sequentially stained for histone H3, followed by 5hmC. The results clearly demonstrate that the 5hmC-enriched regions colocalise with H3K4me2, a typical euchromatin mark (Fig. 3a). The H3K4me2 pattern on the largest mouse chromosome, Chr1, overlapped with 5hmC and with mouse R-bands (Fig. 3b, c). The H3K4me2 pattern obtained in TKO ESCs was similar to the R-band pattern, but was opposite to the G-band pattern (Supplement S3), and suggests that the euchromatin and heterochromatin banding pattern is independent of 5hmC and 5mC. Thus, 5hmC accumulation in euchromatic regions might be regulated by both Tet accessibility and preexisting 5mC pools.

**Fig. 4:**
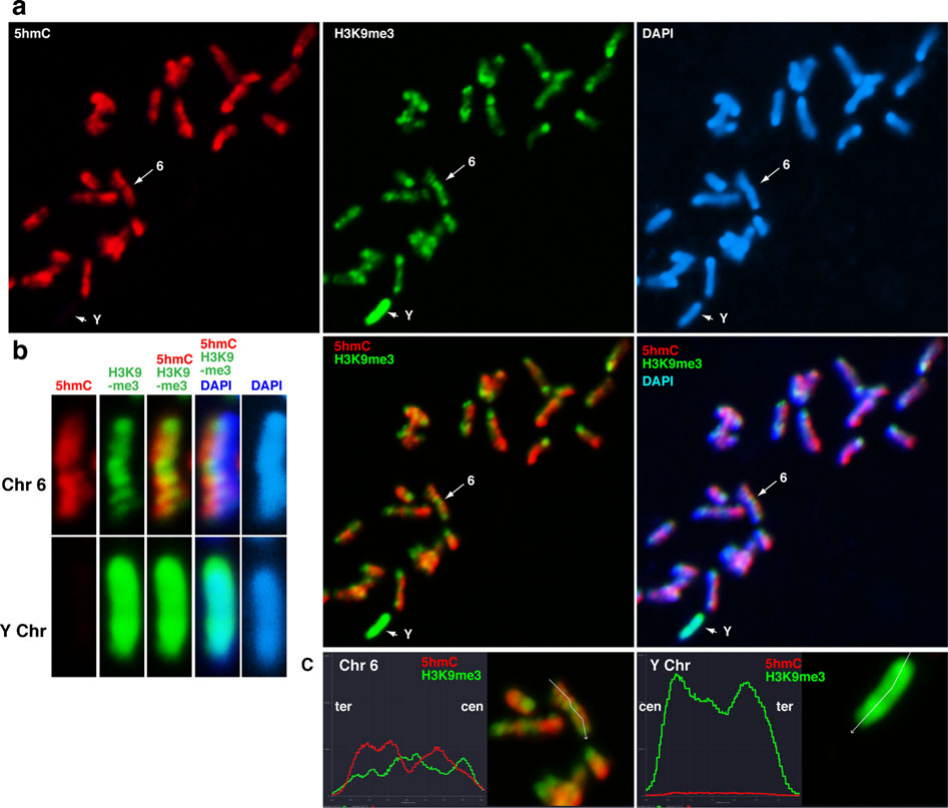
Negative correlation between 5hmC and H3K9me3. **a** Metaphase chromosomes prepared by spin preparation were co-immunostained for 5hmC (*red*) and H3K9me3 (*green*), a heterochromatin mark. The *red* and *green* bands did not overlap in mouse ESCs. **b** This was also shown by an inverse distribution of two-colour fluorescence through the long axis of Chr6. H3K9me3 was distributed uniformly across Y (*arrowheads* in **a**), while H3K9me3 was only enriched in G-bands on other chromosomes, including Chr6 (*arrows*), as determined by anti-H3K9me3 staining. **c** Profiles of the flourescent intensity values for 5hmC and H3K9me3 along the lines drawn across the long axis of Chr6 and Y

**Fig. 5:**
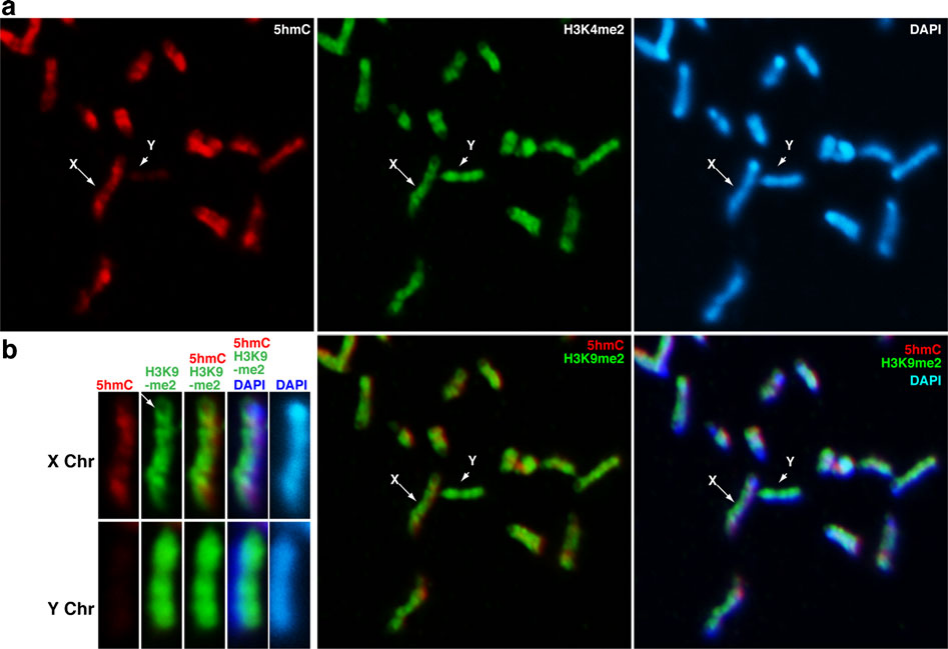
No correlation was observed between 5hmC and H3K9me2, a heterochromatin mark. **a** Metaphase chromosomes prepared by spin preparation were sequentially stained for H3K9me2 (*green*) and 5hmC (*red*). Neither was it present on pericentric heterochromatin. These regions lacked 5hmC, as shown on X (**b** and *arrows* in **a**). H3K9me2 is uniformly distributed across the Y chromosome (*arrowheads* in **a**)

5hmC was excluded from perinuclear and intra-nuclear heterochromatin that were strongly stained by DAPI (Supplement S4). Perinuclear heterochromatin in ESCs is less abundant than in somatic cells, where major and minor satellite sequences accumulate, followed by H3K9me3 and HP1 recruitment (Maison et al. 2011). To identify heterochromatic regions, chromosomes were stained with anti-H3K9me3 antibody. We found that H3K9me3 signals matched the G-band pattern in mouse ESCs (Fig. 4a). H3K9me3 was strongly enriched at the pericentric regions and the Y chromosome. Mouse Chr6 showed an inverse relationship between 5hmC and H3K9me3 (Fig. 4b, c). It has been shown that centromeric function is stably maintained by a combination of H3K9me3 and HP1 in a 5mC-independent manner in TKO ESCs (Kobayakawa et al. 2007). Thus, the exclusion of Tet enzymatic function from pericentric heterochromatin may not be critical for stabilising centromeric function through 5mC maintenance. In addition, H3K9me2, an inactive mark, is broadly associated with both euchromatin and heterochromatin in mouse chromosomes (Fig. 5a). On the Y chromosome, H3K9me2 and H3K9me3 displayed similar patterns (Fig. 5b). Although H3K9me2 was more abundant in G-bands, it was excluded from pericentric satellite repeats (Fig. 5b, arrow). Therefore, H3K9me2 is not positively or negatively correlated with 5hmC in mouse ESCs.

**Fig. 6:**
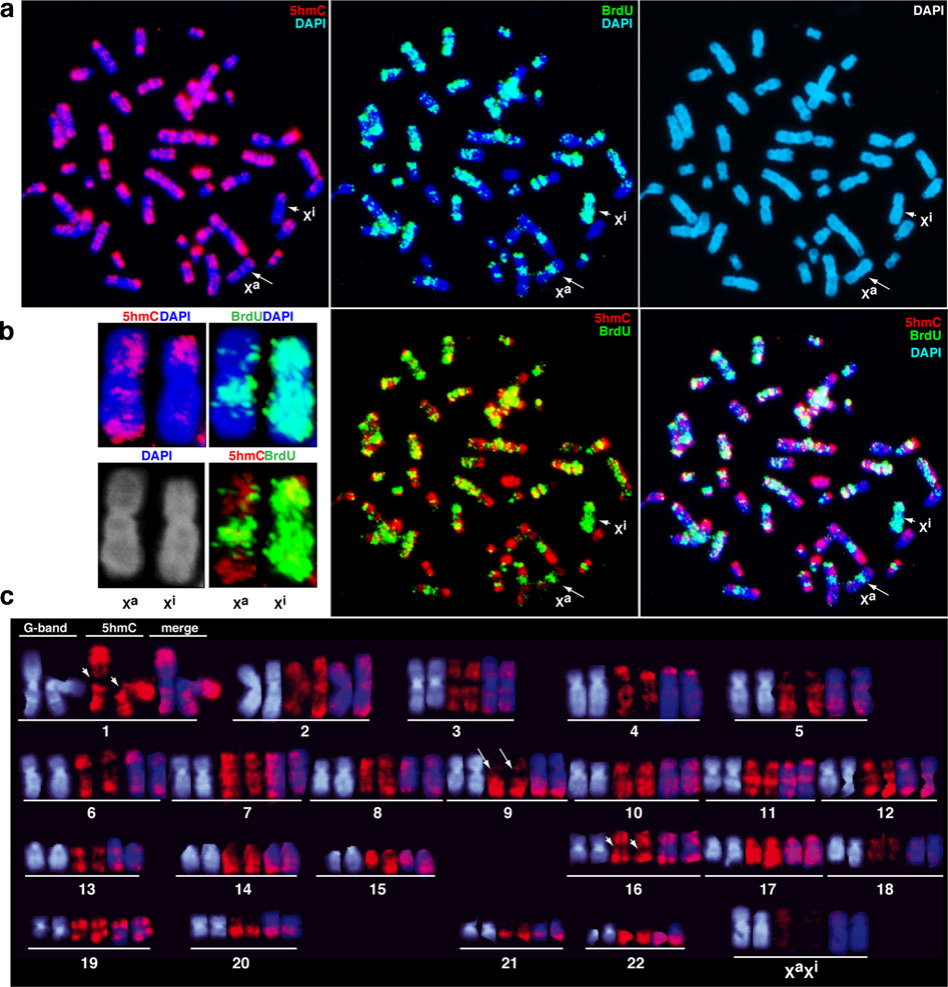
Negative correlation between 5hmC and facultative heterochromatin on human inactive X chromosome (Xi). **a** Metaphase chromosomes were collected from differentiated hiPSCs after the incorporation of BrdU during late S. Xi (*arrowheads*) was identified as entirely BrdU-positive chromosome (*green*). The Xi generally did not contain 5hmC (*red*), excluding the pseudoautosomal regions located at the end of the short arm of Xs. Active X chromosome (Xa) was more hydroxymethylated (*arrow*). **b** The difference in 5hmC and BrdU staining between Xa and Xi is shown. **c** In human cells, the 5hmC pattern was generally inverse that of G-bands. Remarkably, 5hmC was absent in the proximal regions of long arm of human Chr1 and Chr16 (*arrowheads*), and Chr9 (*arrows*), which are known as satellite 2 and 3, respectively

Finally, we analysed 5hmC distribution across human chromosomes in female diff-hiPSCs and investigated the relationship between 5hmC and facultative heterochromatin of the inactive X chromosome (Xi). In mammals, one of two homologous X chromosomes is randomly inactivated in female somatic cells for gene dosage compensation. Mammalian Xi can be characterised by a number of aspects: late replication, absence of H3K4me2, H3K9ac, and H4Kac, and enrichment of H3K9me2, H3K27me3, H4K20me1, H2AK119ub, and macroH2A (Koina et al. 2009). After BrdU incorporation in late S, we were able to easily distinguish Xi from a homologous pair of X chromosomes (Fig. 6a, b). Interestingly, 5hmC was not well distributed on Xi. Human X contains pseudoautosomal regions on both ends of the short and long arms, PAR1 and PAR2, respectively (Flaquer et al. 2008). PAR1, which has been known to escape X inactivation, was strongly enriched with 5hmC on both Xs (Fig. 6b). The reduced distribution of 5hmC on Xi suggests that Tet enzymes are excluded from facultative heterochromatin in a DNA sequence-independent manner. However, 5hmC levels were low on both Xs rather than on autosomes since it has been reported that low levels of 5mC are present on the X and Y chromosomes in primates (Bernardino et al. 2000). In addition, human genomes contain heterochromatic blocks called satellite 2 and 3 on human Chr1, 2, 9, 10, and 16 (Tagarro et al. 1994). As expected, 5hmC did not accumulate at the satellite 2 on human Chr1 and Chr16, and at the satellite 3 on human Chr9 (Fig. 6c).

## Discussion

This report describes that the localisation pattern of 5hmC was completely opposite to that of H3K9me3 and G-bands, but was similar to that of H3K4me2/3 and R-bands on mouse and human chromosomes. In addition, we demonstrated that H3K9me2 was not correlated with 5hmC as H3K9me2 was more abundant in G-bands but excluded from pericentric satellite repeats. The evidence that Tet enzymes target fully methylated and hemimethylated DNA (Ficz et al. 2011; Pastor et al. 2011; Tahiliani et al. 2009) suggests that euchromatin is enriched with preexisting 5mC. Therefore, Tet enzymes might reduce 5mC levels in euchromatin to prevent nonspecific DNA methylation by de novo Dnmt enzymes.

It has become apparent that histone methyltransferases are linked with Dnmt activity. For example, when Suv39h1 and Suv39h2, which catalyse H3K9me2/3 at pericentric heterochromatin and recruit Dnmt3b, are both mutated, cells display an altered DNA methylation profile at pericentric satellite repeats (Lehnertz et al. 2003). In addition, G9a and GLP, which are two related histone H3 methylation enzymes essential for global H3K9me1/2, can recruit Dnmt3a and 3b. Thus, lack of either results in DNA hypomethylation, especially in euchromatin (Epsztejn-Litman et al. 2008; Tachibana et al. 2005, 2008). Moreover, the Polycomb group (PcG) protein Ezh2, a histone methyltransferase that can recruit H3K27me, interacts with Dnmts through PcG complexes (Vire et al. 2006). These histone methyltransferases therefore recruit Dnmts not only to heterochromatin but also to euchromatin. Our results suggest that nonselective methylation is likely refined by Tets based on their target preference, which results in the elimination of 5mC from euchromatic regions. Mechanisms leading to the euchromatin-specific recruitment of Tets remain to be elucidated.

Recent studies have shown that 5mC is selectively converted into 5hmC by Tet3 in paternal pronuclei (patPN), which leads to patPN-specific active demethylation, while 5mC on maternal pronuclei (matPN) is erased in a cell cycle-dependent manner (Gu et al. 2011; Inoue and Zhang 2011; Iqbal et al. 2011; Wossidlo et al. 2011). In mouse zygotes, MatPN maintains H3K9me2 and me3 throughout the first mitotic division just before the two-cell stage. In addition, while MatPN maintains all types of H3 methylation, such as H3K4me1/3, H3K27me2/3, and H4K20me3, patPN remains unmodified until the first DNA replication (Lepikhov et al. 2010). These pieces of evidence show that euchromatin may recruit Tet enzymes to patPN as a default state. Recently, it has been shown that PGC7 binding to the MatPN genomes marked with H3K9me2 protect 5mC to 5hmC conversion in mouse zygotes (Nakamura et al. 2012; Wossidlo et al. 2011). However, while PGC7 is only expressed in zygotes, ESCs and PGCs, it is never expressed in somatic cells (Saitou et al. 2002). Moreover, we have shown that H3K9me2 is excluded from pericentric satellite repeats. Thus, it is expected that PGC7 does not participate in the R-banded 5hmC patterns in general, though H3K9me2 might inhibit 5mC to 5hmC conversion in euchromatin at gene level. Thus, we conclude that preexisting H3K9me3 levels influence Tet enzyme accessibility at chromosome levels.

Another possibility is that Tet enzymatic activity might also be regulated in a cell cycle-dependent manner. The Tet3 enzyme may be temporally activated during the early stage of zygotic development and then immediately inactivated before paternal and maternal chromosomes are combined at the first mitosis. This conjecture is supported by the observation that additional conversion of 5mC to 5hmC is never induced on maternal chromosomes before at least the eight-cell stage (Inoue et al. 2011; Inoue and Zhang 2011). Recently, it was shown that Ezh2 could be phosphorylated by the cyclin-dependent kinase CDK1, which phosphorylates the threonines of Ezh2, followed by Ezh2 ubiquitination and degradation by the proteasome (Wu and Zhang 2011). Tets may also be regulated in a similar way. Moreover, paternal-specific 5mC to 5hmC conversion might be explained by asynchronous replication between patPN and matPN, as some reports suggest that patPN initiates DNA replication earlier than matPN by up to 2 h (Yamauchi et al. 2009), although this observation remains to be verified. Thus, there is a possibility that euchromatin-specific conversion of 5mC to 5hmC is regulated by a combination of cell cycle-dependent chromatin decondensation and Tet enzymatic instability. In contrast to the situation in zygotes, cyclic restoration of 5mC by de novo Dnmts is necessary after 5hmC-mediated demethylation through cell divisions especially at euchromatin in mouse ESCs and human diff-iPSCs as every cell always showed clear R-banded patterns after 5hmC antibody staining. Thus, we suggest that the balance of two enzymatic activities, Tets and de novo Dnmts, regulates not only the levels of 5mC status in mitotic cells but also the intra-chromosomal localisation of 5mC in heterochromatin by the euchromatin-specific conversion of 5mC to 5hmC on the basis of high-order chromatin structure that is defined by preexisting histone modifications and DNA sequences.

## Electronic supplementary material

Below is the link to the electronic supplementary material.

Supplement S1 Comparative analysis of the link between 5hmC localisation and replication bands. Chromosome pairs stained with DAPI for G-bands (blue) and anti-5hmC (red) and anti-BrdU (green) in mouse J1 ESCs were arranged side-by-side. The merged images of 5hmC and BrdU revealed an inverse relationship on every mouse chromosome. X, X chromosome; Y, Y chromosome. (JPEG 71 kb)

High resolution image (TIFF 3351 kb)

Supplement S2 Localisation of repeat sequences on pericentric regions of mouse chromosomes. Fluorescence in situ hybridization signals for mouse Cot1 show that pericentric repeat sequences were denatured well by the two procedures used here. (JPEG 41 kb)

High resolution image (TIFF 2506 kb)

Supplement S3 Localisation of H3K4me2 on mouse chromosomes in TKO ESCs. Metaphase chromosomes prepared by spin preparation were sequentially stained for H3K4me2 (green) and 5hmC (red). H3K4me2 bands, but not 5hmC bands, showed typical R-bands in mouse TKO ESCs. R-banded distribution of H3K4me2 through the long axis of X Chr. (JPEG 25 kb)

High resolution image (TIFF 1153 kb)

Supplement S4 Exclusion of 5hmC from perinucleic and intranucleic heterochromatin in mouse ESCs. A haze redacted image of metaphase chromosomes and nuclei stained with anti-5hmC and DAPI, which shows euchromatin-specific localisation of 5hmC. Scale bar, 10 μm. (JPEG 39 kb)

High resolution image (TIFF 1876 kb)

## Abbreviations

5hmC: 5-Hydroxymethylcytosine
5mC: DNA cytosine methylation
Dnmt: DNA methyltransferase
ESCs: Embryonic stem cells
iPSCs: Induced pluripotential stem cells
MBD: Methyl-binding domain
PGCs: Primordial germ cells
PN: Pronucleus
Tet: Tet (Ten-eleven translocation) methylcytosine dioxygenase
