# Supplementary figures and images for: Chromosome-wide regulation of euchromatin-specific 5mC to 5hmC conversion in mouse ES cells and female human somatic cells
Source: Chromosome Res. 2012 Oct 31;20(7):837–48. doi: 10.1007/s10577-012-9317-9 (PMC3524505; doi:10.1007/s10577-012-9317-9)

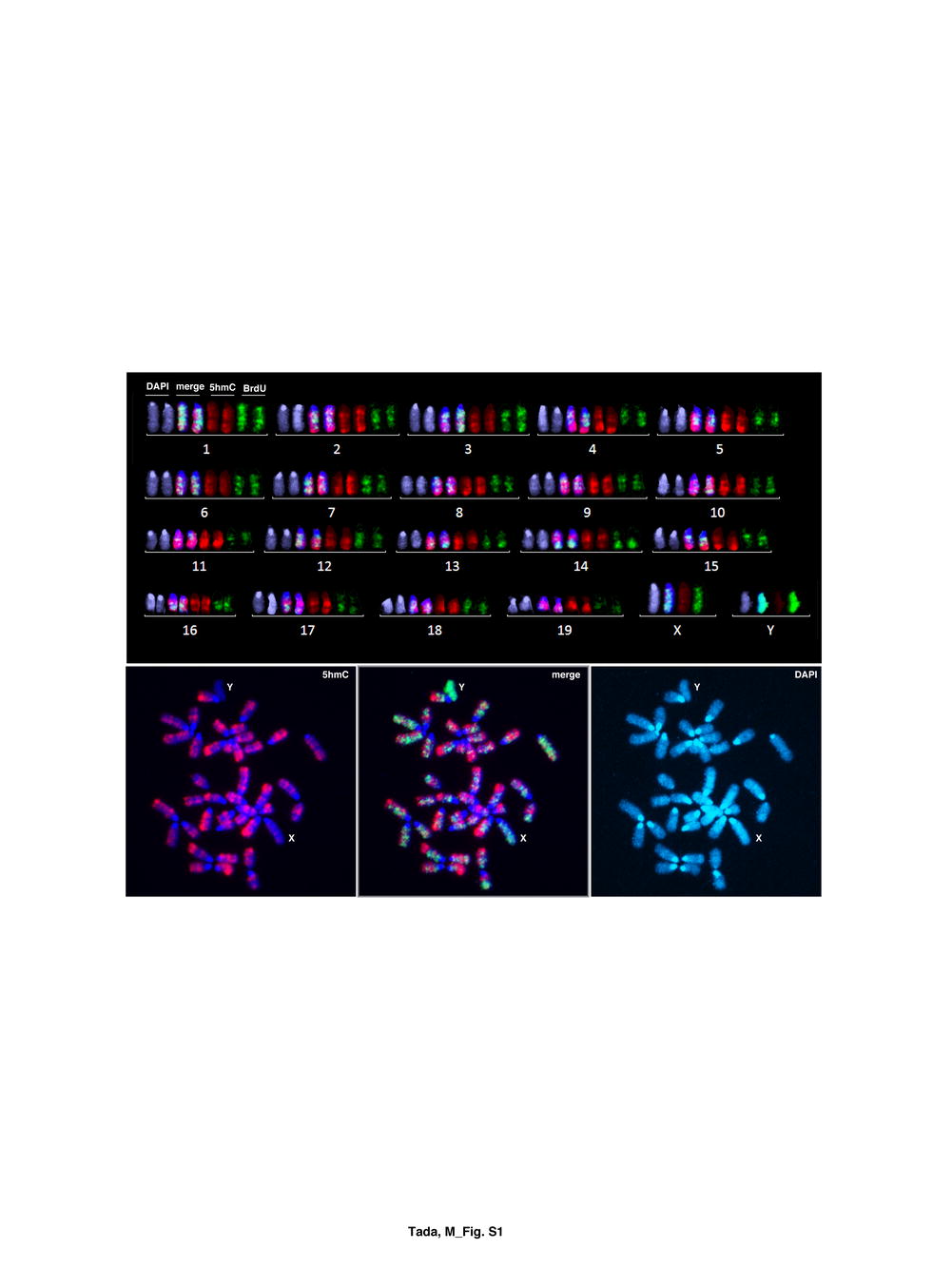

Supplement: Supplementary file 1 — Comparative analysis of the link between 5hmC localisation and replication bands. Chromosome pairs stained with DAPI for G-bands (blue) and anti-5hmC (red) and anti-BrdU (green) in mouse J1 ESCs were arranged side-by-side. The merged images of 5hmC and BrdU revealed an inverse relationship on every mouse chromosome. X, X chromosome; Y, Y chromosome. (JPEG 71 kb) [file 10577_2012_9317_Fig7_ESM.jpg]

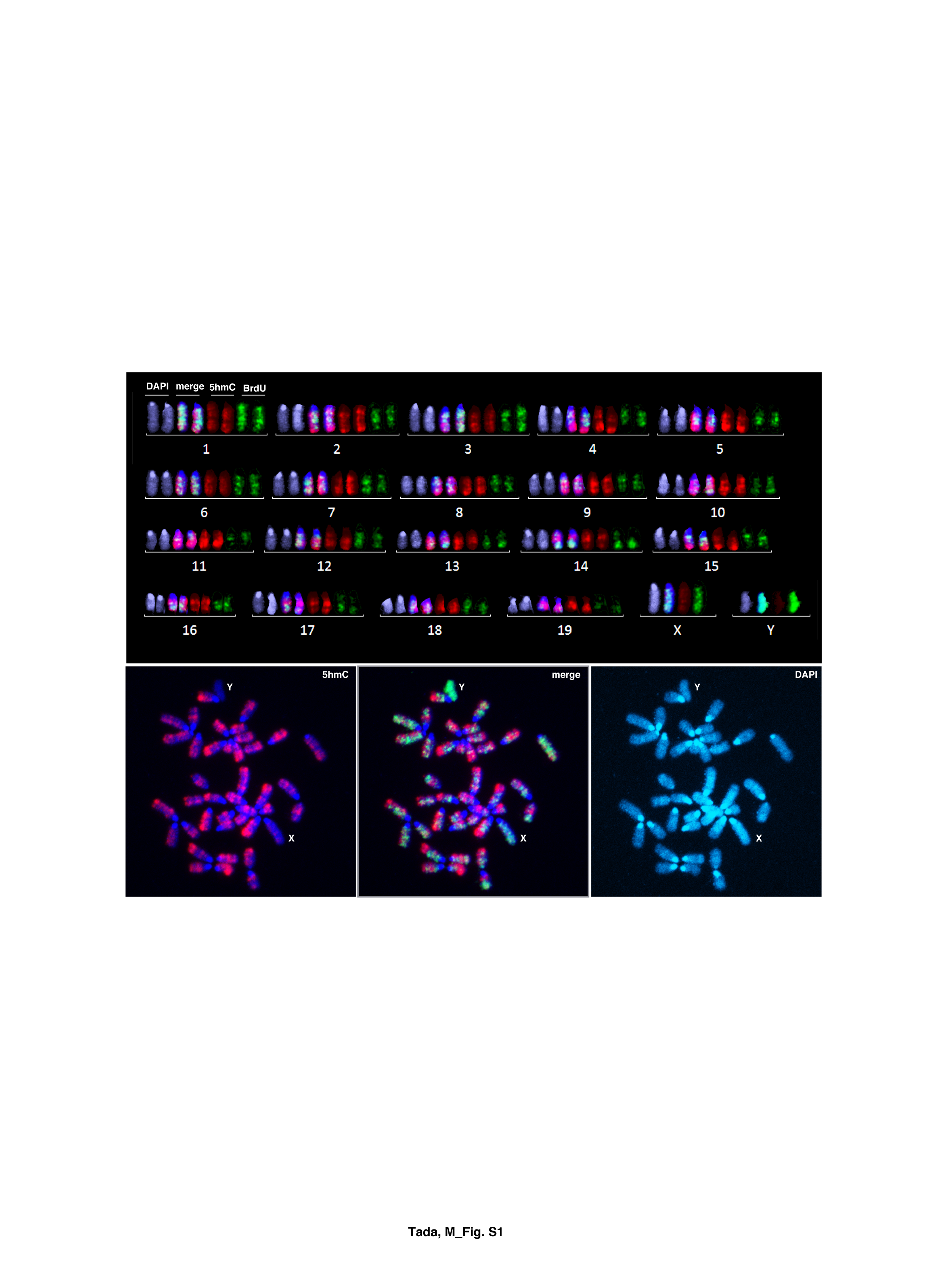

Supplement: Supplementary file 2 — High resolution image (TIFF 3351 kb) [file 10577_2012_9317_MOESM1_ESM.tif]

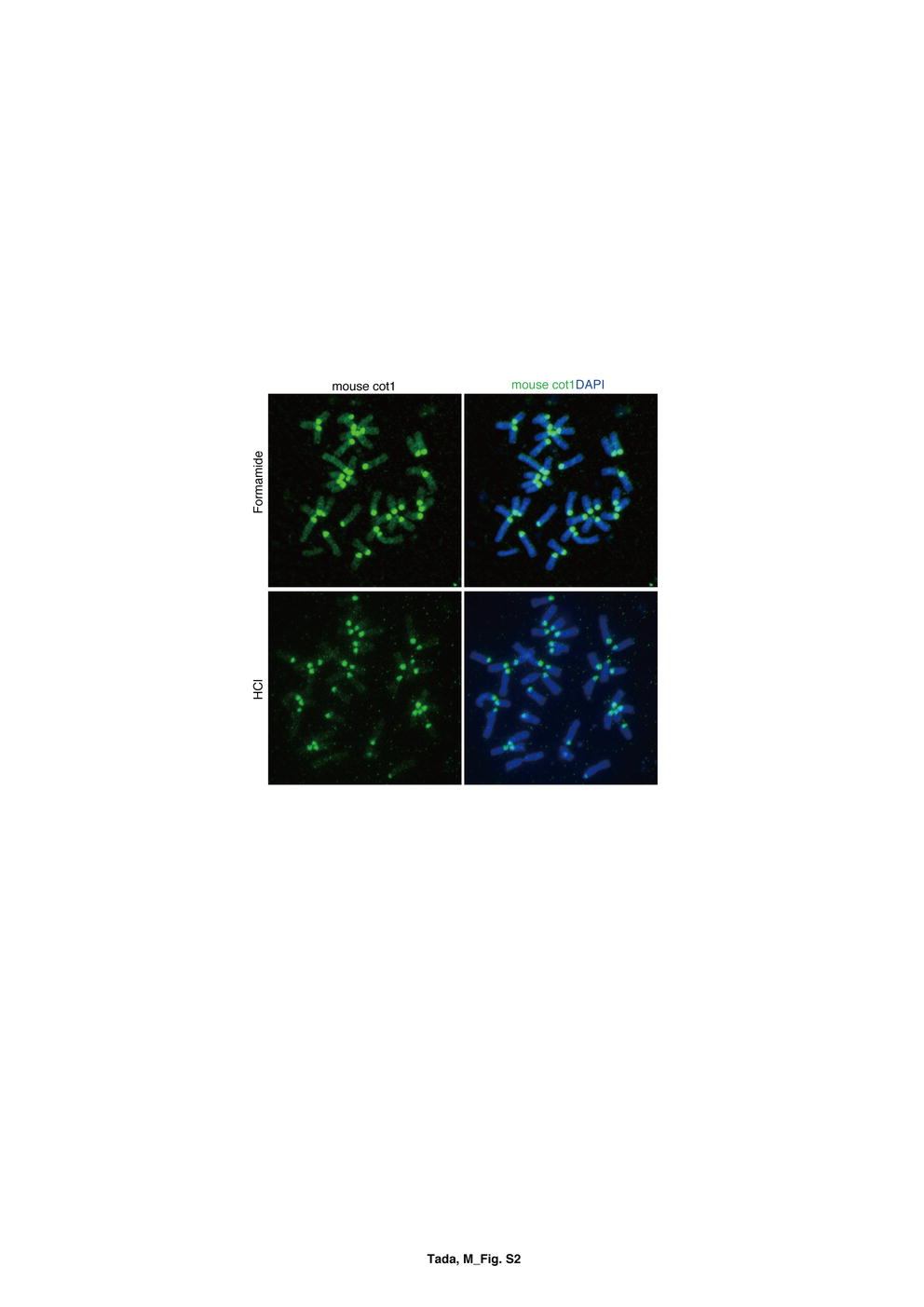

Supplement: Supplementary file 3 — Localisation of repeat sequences on pericentric regions of mouse chromosomes. Fluorescence in situ hybridization signals for mouse Cot1 show that pericentric repeat sequences were denatured well by the two procedures used here. (JPEG 41 kb) [file 10577_2012_9317_Fig8_ESM.jpg]

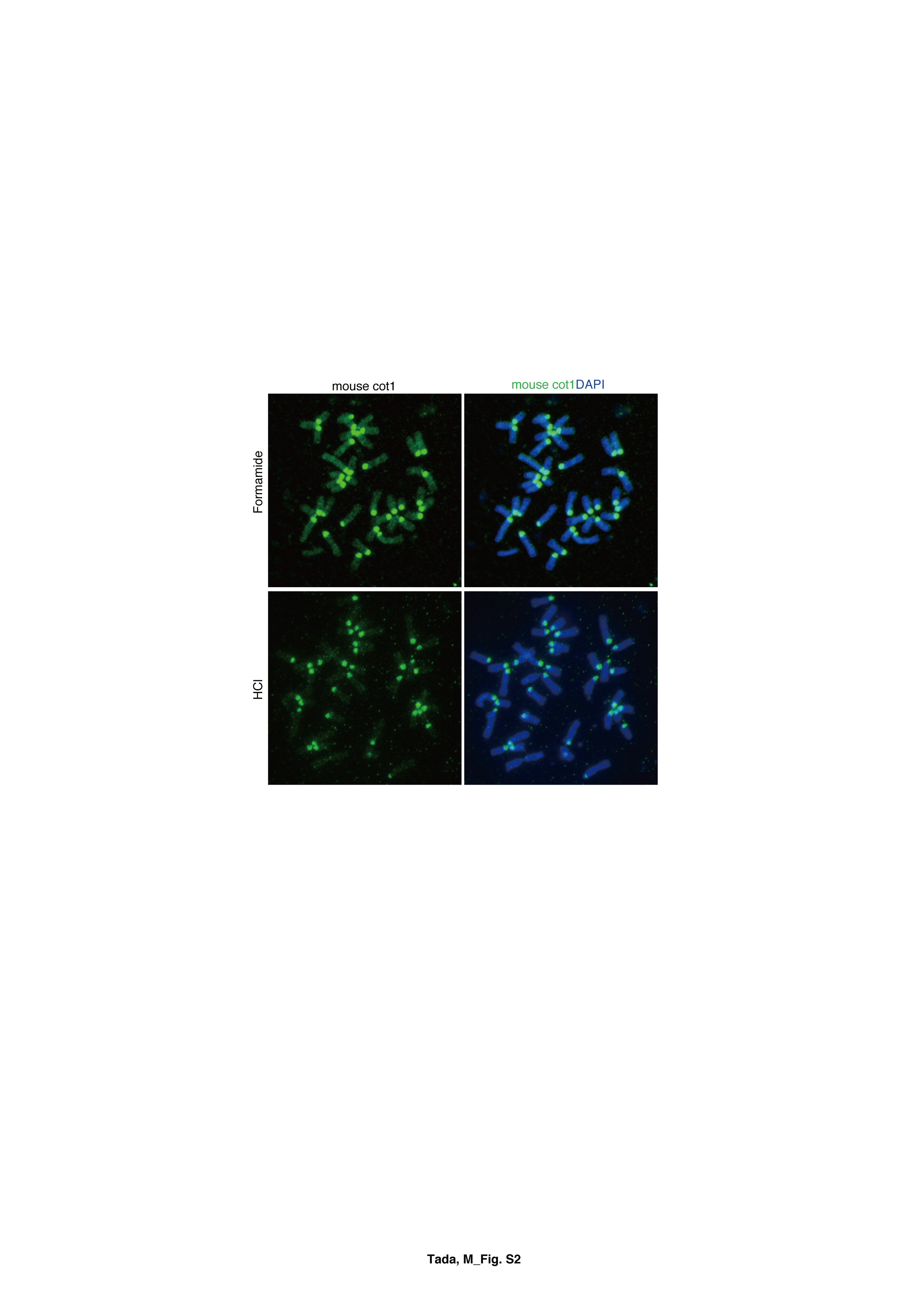

Supplement: Supplementary file 4 — High resolution image (TIFF 2506 kb) [file 10577_2012_9317_MOESM2_ESM.tif]

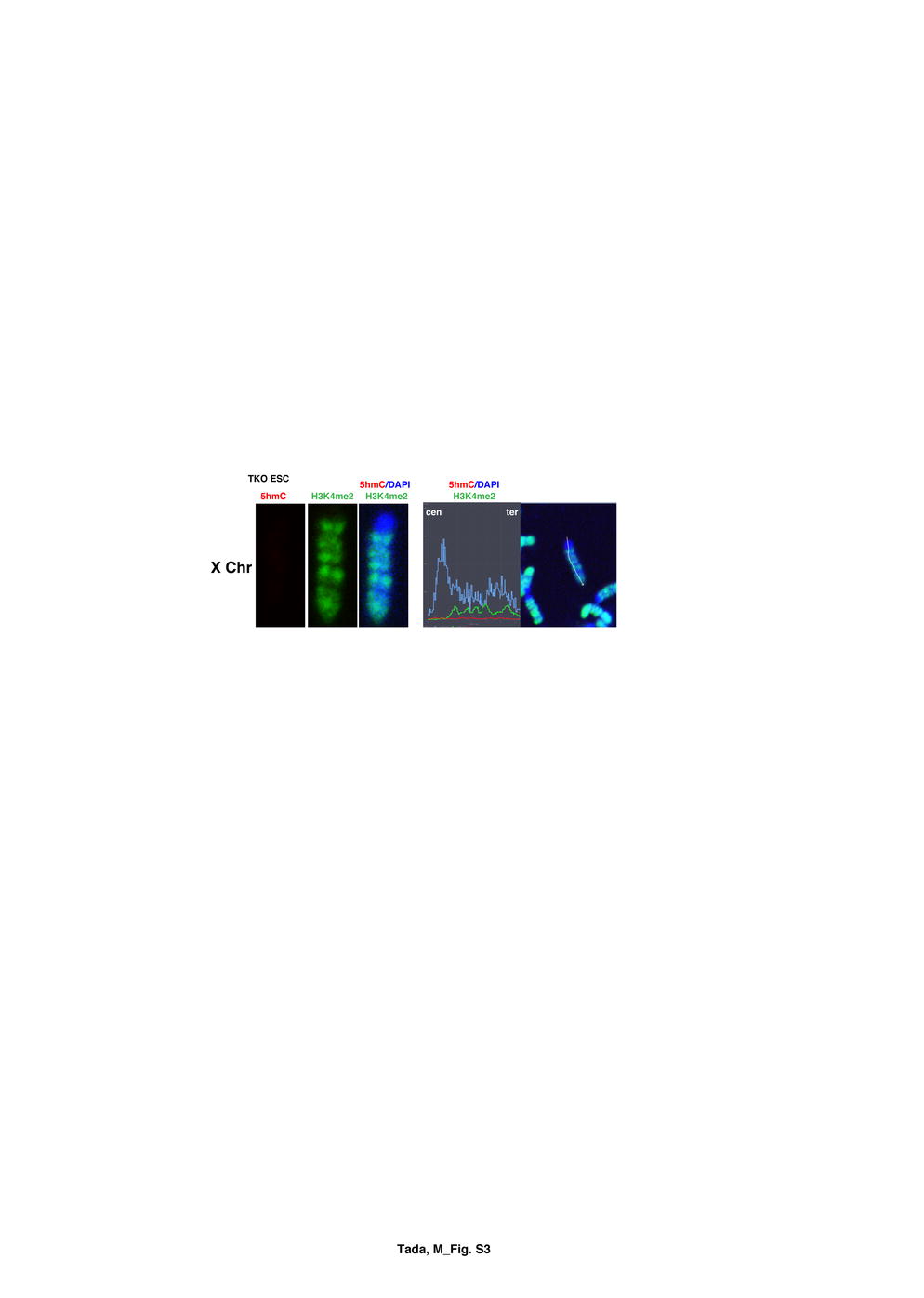

Supplement: Supplementary file 5 — Localisation of H3K4me2 on mouse chromosomes in TKO ESCs. Metaphase chromosomes prepared by spin preparation were sequentially stained for H3K4me2 (green) and 5hmC (red). H3K4me2 bands, but not 5hmC bands, showed typical R-bands in mouse TKO ESCs. R-banded distribution of H3K4me2 through the long axis of X Chr. (JPEG 25 kb) [file 10577_2012_9317_Fig9_ESM.jpg]

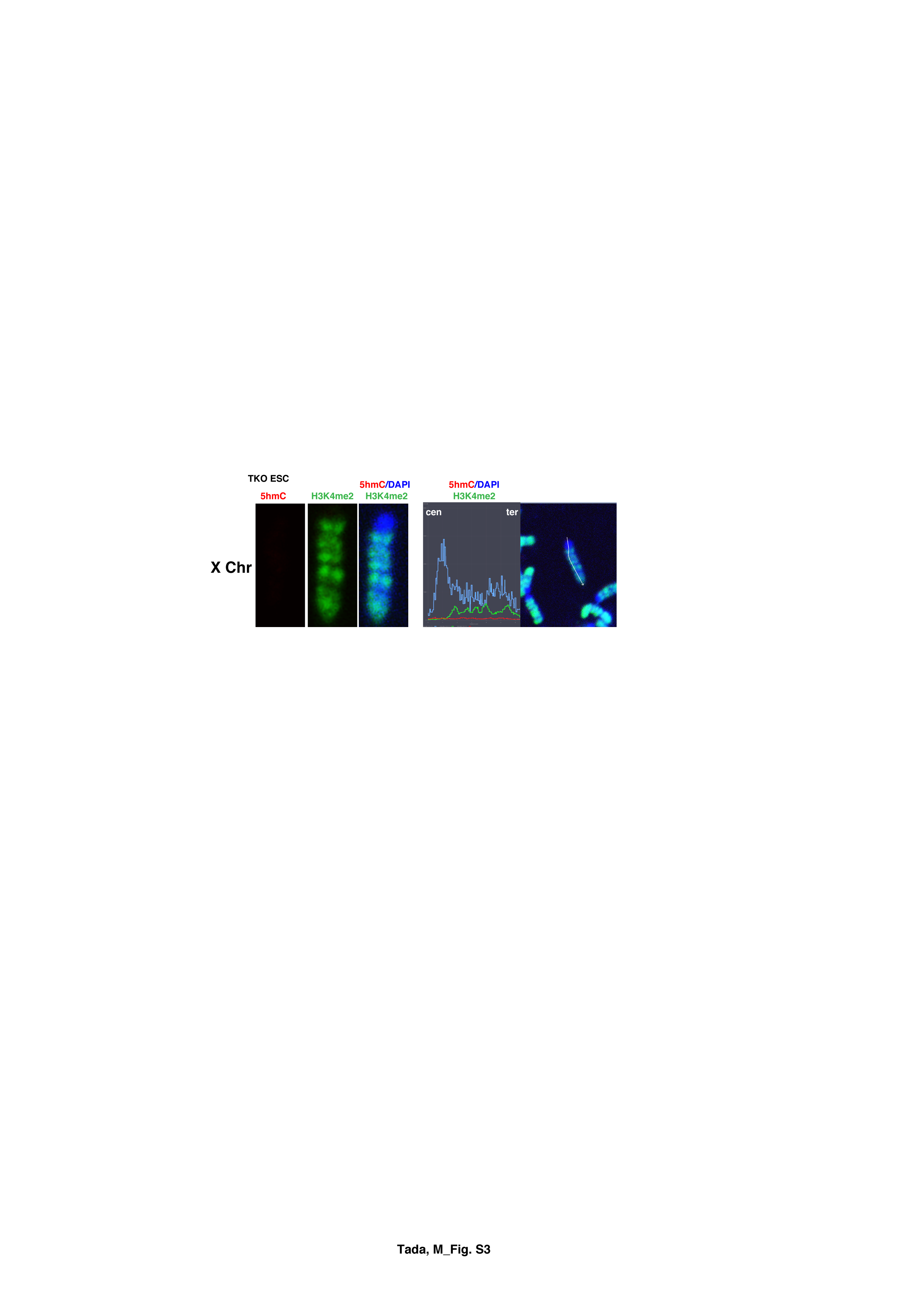

Supplement: Supplementary file 6 — High resolution image (TIFF 1153 kb) [file 10577_2012_9317_MOESM3_ESM.tif]

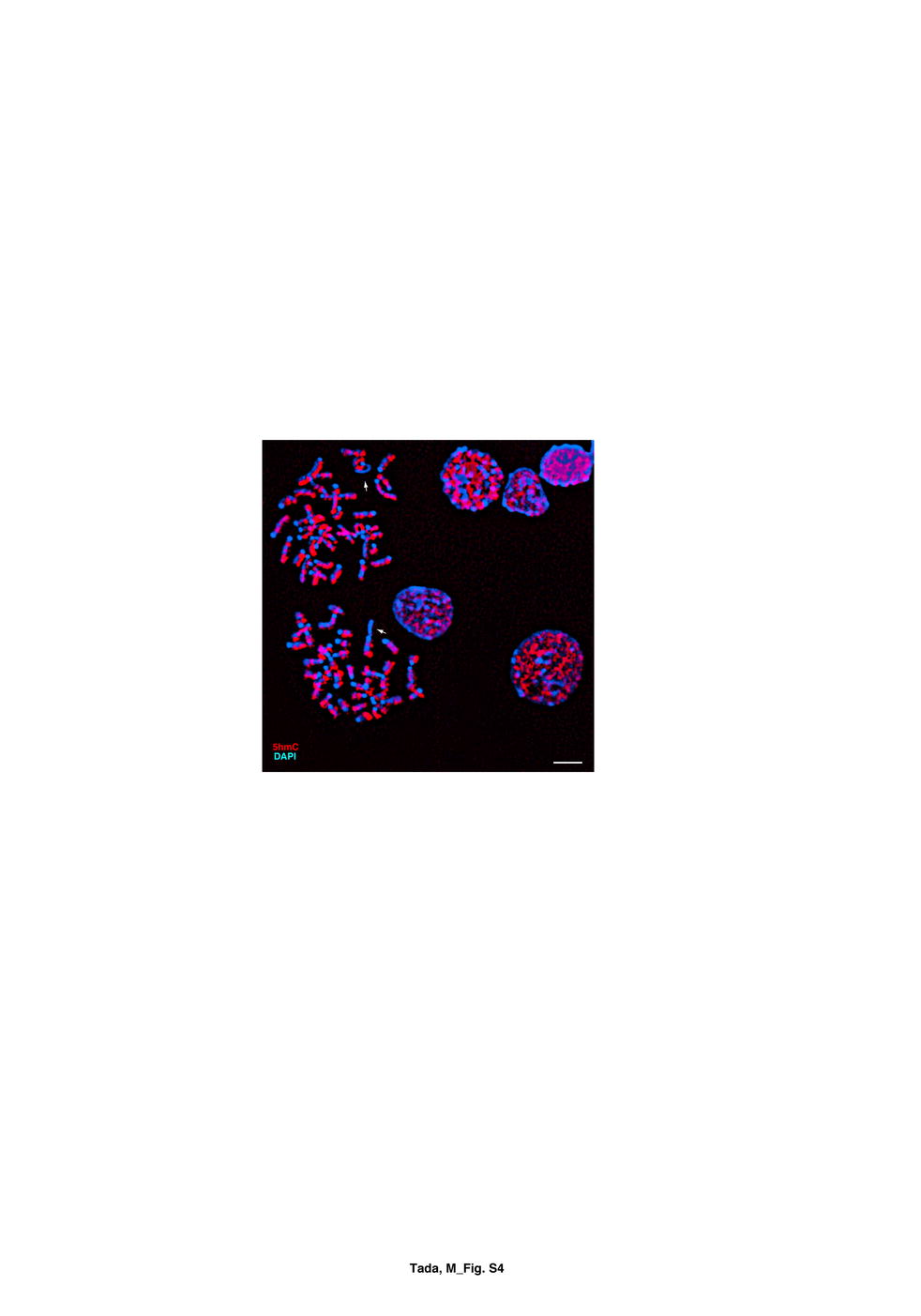

Supplement: Supplementary file 7 — Exclusion of 5hmC from perinucleic and intranucleic heterochromatin in mouse ESCs. A haze redacted image of metaphase chromosomes and nuclei stained with anti-5hmC and DAPI, which shows euchromatin-specific localisation of 5hmC. Scale bar, 10 μm. (JPEG 39 kb) [file 10577_2012_9317_Fig10_ESM.jpg]

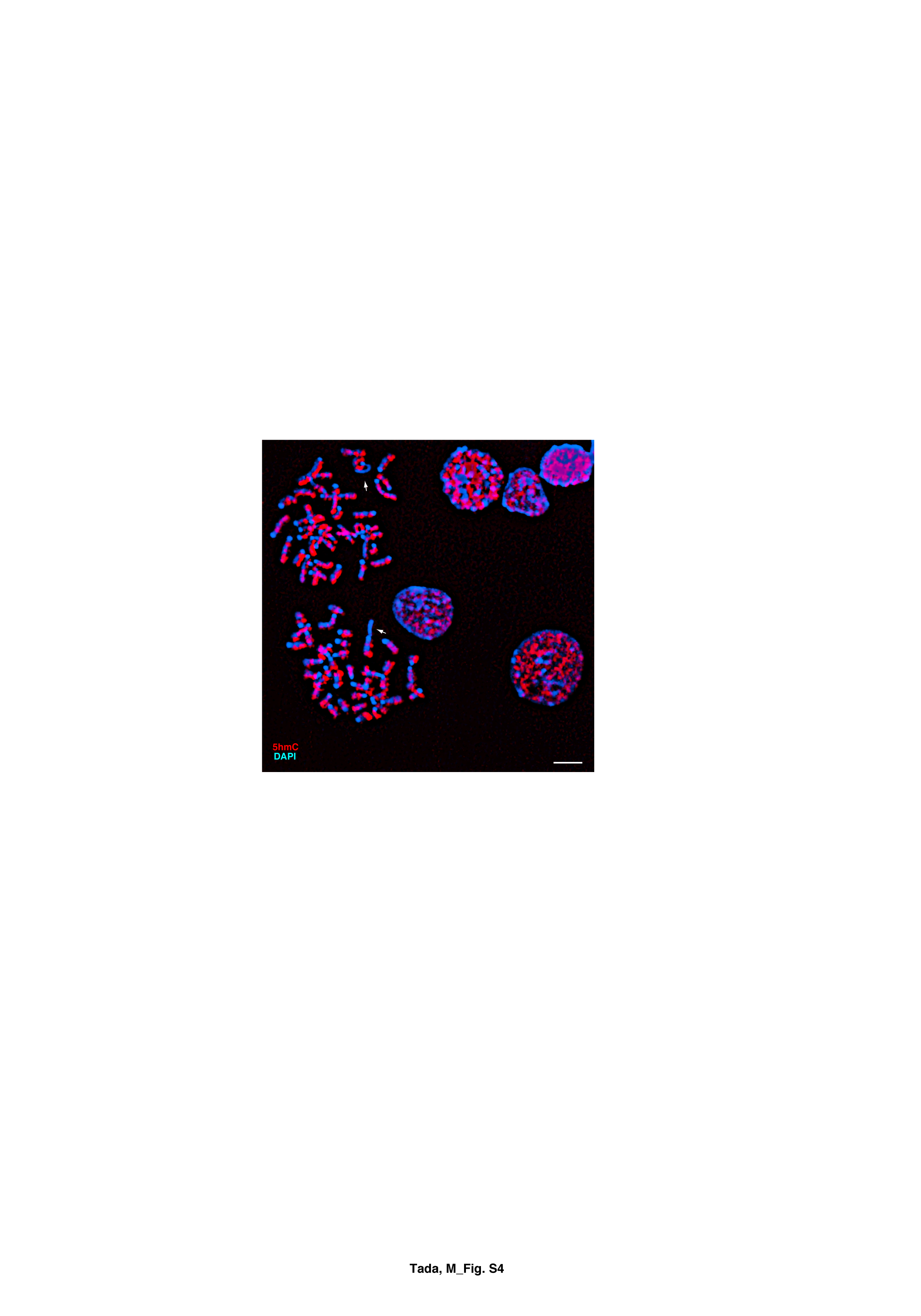

Supplement: Supplementary file 8 — High resolution image (TIFF 1876 kb) [file 10577_2012_9317_MOESM4_ESM.tif]
